# Inhibition of ADAM10 promotes the clearance of Aβ across the BBB by reducing LRP1 ectodomain shedding

**DOI:** 10.1186/s12987-016-0038-x

**Published:** 2016-08-08

**Authors:** B. Shackleton, F. Crawford, C. Bachmeier

**Affiliations:** 1 The Roskamp Institute, Sarasota, FL 34243 USA; 2 The Open University, Milton Keynes, Buckinghamshire MK7 6AA UK

**Keywords:** Alzheimer’s disease, A desintegrin and metalloproteinase domain containing protein 10 (ADAM10), Low density lipoprotein receptor-related protein 1 (LRP1), Blood–brain barrier (BBB), Beta-amyloid (Aβ)

## Abstract

**Background:**

Transport across the blood–brain barrier (BBB) is an important mediator of beta-amyloid (Aβ) accumulation in the brain and a contributing factor in the pathogenesis of Alzheimer’s disease (AD). One of the receptors responsible for the transport of Aβ in the BBB is the low density lipoprotein receptor-related protein 1 (LRP1). LRP1 is susceptible to proteolytic shedding at the cell surface, which prevents endocytic transport of ligands. Previously, we reported a strong inverse correlation between LRP1 shedding in the brain and Aβ transit across the BBB. Several proteases contribute to the ectodomain shedding of LRP1 including the α-secretase, a desintegrin and metalloproteinase domain containing protein 10 (ADAM10).

**Methods:**

The role of ADAM10 in the shedding of LRP1 and Aβ BBB clearance was assessed through pharmacological inhibition of ADAM10 in an in vitro model of the BBB and through the use of ADAM10 endothelial specific knock-out mice. In addition, an acute treatment paradigm with an ADAM10 inhibitor was also tested in an AD mouse model to assess the effect of ADAM10 inhibition on LRP1 shedding and Aβbrain accumulation.

**Results:**

In the current studies, inhibition of ADAM10 reduced LRP1 shedding in brain endothelial cultures and increased Aβ42 transit across an in vitro model of the BBB. Similarly, transgenic ADAM10 endothelial knockout mice displayed lower LRP1 shedding in the brain and significantly enhanced Aβ clearance across the BBB compared to wild-type animals. Acute treatment with the ADAM10-selective inhibitor GI254023X in an AD mouse model substantially reduced brain LRP1 shedding and increased Aβ40 levels in the plasma, indicating enhanced Aβ transit from the brain to the periphery. Furthermore, both soluble and insoluble Aβ40 and Aβ42 brain levels were decreased following GI254023X treatment, but these effects lacked statistical significance.

**Conclusions:**

These studies demonstrate a role for ADAM10 in the ectodomain shedding of LRP1 in the brain and the clearance of Aβ across the BBB, which may provide a novel strategy for attenuating Aβ accumulation in the AD brain.

## Background

Alzheimer’s disease is the most common cause of dementia in the elderly and is characterized, in part, by the deposition of amyloid beta (Aβ) in the brain and cerebrovasculature. The accumulation of soluble Aβ precedes the initial neurodegeneratitive and neurotoxic cascade and correlates strongly with reductions in cognitive performance [1–3]. It has been reported that the accumulation of Aβ in AD is primarily the result of inadequate Aβ elimination, with AD patients showing a 30 % reduction in the overall clearance of Aβ from the brain [4, 5]. Furthermore, in the Dutch-type mouse model of AD, the elimination of Aβ through the BBB is significantly reduced, which results in Aβ deposition along the cerebral vessels and the development of cerebral amyloid angiopathy (CAA) [6, 7].

One of the prominent mechanisms responsible for the elimination of Aβ from the brain involves transcytosis across the blood–brain barrier (BBB). The rate in which Aβ is cleared from the brain to the blood through the BBB is greater than the rate in which it can be removed via interstitial fluid bulk flow [8], likely due to the presence of various transport systems including the low density lipoprotein receptor-related protein 1 (LRP1) [9–11]. After binding to LRP1 at the abluminal surface of the brain endothelium, Aβ is transported across the BBB where it is then released into the general circulation [12, 13]. Recently, conditional endothelial LRP1 KO mice developed by Storck et al. [11] showed significant reductions in the transit of Aβ across the BBB and deficits in memory and spatial learning, highlighting the importance of BBB transport in the elimination of Aβ from the brain.

In addition to the membrane-associated protein, LRP1 also exists in a soluble form as a result of proteolytic cleavage at the surface of the cell. This soluble LRP1 fragment retains its binding capacity, but loses its ability to endocytose and transport ligands [14–16]. In line with this, recent work by our group demonstrated a strong inverse correlation between LRP1 shedding in the brain and Aβ clearance across the BBB [17], indicating that reductions in brain LRP1 shedding could promote Aβ clearance across the BBB and attenuate Aβ accumulation in the AD brain. As such, investigating the factors that regulate LRP1 shedding in the brain may provide therapeutic opportunities to lower Aβ burden and modulate the AD phenotype. One of the enzymes implicated in LRP1 ectodomain shedding is the α-secretase, ADAM10 (a desintegrin and metalloproteinase domain containing protein 10), [18]. The following studies examined the influence of ADAM10 on LRP1 shedding in vitro and in vivo and the evaluated the impact of ADAM10 modulation on Aβ clearance across the BBB.

## Methods

### Animals

ADAM10 endothelial KO mice were kindly provided, by Dr. Carl Blobel (Hospital for Special Surgery, New York, NY), Dr. B. De Strooper (VIB Center for the biology of Disease, Leuven, Belgium) and Dr. P. Saftig (Institute of Biochemistry, CAU Kiel, Germany). These mice were generated by crossing ADAM10 flox/flox mice [19] with Tie2-Cre transgenic mice that use the endothelial specific promoter Tie2 to drive the expression of Cre recombinase [20] to produce ADAM10 flox/flox/Tie2-Cre mice. The ADAM10 endothelial KO mice lack ADAM10 expression in the endothelial cells [21]. Wild Type mice on a C57BL/6 background were used as control animals. To evaluate the effect of ADAM10 modulation in an animal model of AD, we utilized transgenic mice overexpressing the human APP695 sw mutation and the presenilin-1 mutation M1 46L (PSAPP) which results in the overproduction of human Aβ [22]. All mice were group housed in a temperature and humidity controlled environment on a 12 h light/dark cycle with free access to food and water. All experiments involving animals were approved by the Institutional Animal Care and Use Committee of the Roskamp Institute.

### Preparation of Aβ42 peptides

Using a standard protocol to limit aggregation, both human recombinant Aβ42 and fluorescein labeled Aβ42 were solubilized in 1,1,1,3,3,3-hexafluoro-2-propanol to acquire a monomeric/dimeric sample and minimize the formation of β-sheet structures as we previously described [23].

### In vitro LRP1 shedding

Human Brain Microvessel endothelial cells (HBMECs) (ScienCell, USA) were seeded at 50,000 cells per cm^2^ onto fibronectin-coated 6-well plates as previously described by our group [24]. When approximately 90 % confluent, cells were treated with Aβ42 (2 μM) or both Aβ42 and the ADAM10-selective inhibitor GI254023X (1 μM) (Sigma-Aldrich, USA) and incubated at 37 °C for 48 h. Our previous work demonstrated that 2 μM Aβ significantly induced LRP1 shedding in the absence of any overt cellular toxicity [25]. After the 48 h incubation, the extracellular media was collected and the level of soluble LRP1 was assessed using an ELISA for LRP1 (Cat# SEB010Hu, Cedar Lane Labs, USA).

### Aβ transit across an in vitro BBB model

The impact of ADAM10 on Aβ42 transcytosis was assessed using an in vitro BBB model previously described by our group [24]. HBMECs were seeded onto fibronectin-coated 24-well membrane inserts forming a polarized monolayer representing the BBB. Aβ transcytosis across the BBB was assessed by exposing the basolateral side (“brain”) to 2 μM fluorescein-Aβ42 (rPeptide, USA) and treating the apical side (“blood”) with the ADAM10-selective inhibitor GI254023X (0.1–1 μM). After 60 min of incubation at 37 °C, samples were collected from the apical compartment to assess the basolateral-to-apical transcytosis of fluorescein-Aβ across the BBB model. To ensure Aβ42 or GI254023X treatment did not disrupt the integrity of the BBB monolayer, we examined the effect of Aβ and GI254023X on the permeability of the paracellular marker, 10 kDa Lucifer yellow-dextran.

### Brain LRP1 shedding and Aβ BBB clearance in vivo

To determine the role of ADAM10 in the clearance of Aβ42 from the brain to the periphery, we examined the appearance of human Aβ42 in the plasma after intracranial Aβ administration as previously described by our group [24, 26]. Briefly, wild-type and ADAM10 endothelial KO mice (16–24 weeks of age) were anesthetized via inhalation using a 3 % isoflurane/oxygen mix. While under anesthesia, 3 μl of human Aβ42 (1 mM) was bilaterally injected into the caudate putamen of the brain (0.5 mm anterior to the bregma and 2 mm lateral to the midline at a depth of 3 mm below the skull surface). Ten minutes after the intracerebral injections, the mice were euthanized and the plasma and brain tissue were collected. Plasma samples were analyzed for human Aβ42 using an ELISA (Invitrogen, USA). Mouse brains were homogenised in 12 ml of ice cold Hanks Balances Salt Solution (HBSS) with a Dounce Homogenizer. For collection of the soluble brain fraction (i.e., non cell-associated), samples were centrifuged at 6000*g* for 15 min to remove intact cells, cell associated proteins and non-soluble components. The levels of LRP1 in the soluble fraction were measured in the supernatant using a LRP1 ELISA (Cat# SEB010Hu, Cedar Lane Labs, USA) and normalized to the total protein content in the brain homogenate as determined by the bicinchoninic acid (BCA) protein assay (Thermo Scientific, USA). Soluble LRP1 levels were expressed as ng of LRP1 per mg protein.

### Aβ, soluble LRP1 and sAPPα levels in PSAPP mice following ADAM10 inhibition

PSAPP mice at 35 weeks of age were injected intraperitoneally with 200 mg/kg of GI254023X or vehicle (DMSO) once per day for 5 consecutive days. At this age, PSAPP mice display elevated levels of Aβ peptides in the brain [22]. This treatment regime has previously been shown to effectively inhibit ADAM10 activity in mice and was well tolerated with no adverse effects reported [27]. One hour after the final injection, the mice were euthanatized and plasma and brain tissue were collected. The right hemisphere was used to examine the levels of soluble and insoluble Aβ40 and Aβ42 in the brain. Briefly, the hemispheres were homogenized by sonication in 700 μl lysis buffer [M-PER + 1 % EDTA +0.2 % PMSF (Thermo Scientific, USA)] supplemented with Halt protease and phosphatase inhibitor cocktail (Thermo Scientific, USA) on ice before centrifugation at 14000*g* for 30 min at 4 °C. 100 μl of the resulting supernatant was mixed with 5 M guanidine in TRIS buffer resulting in the guanidine soluble (GS) fraction. For the guanidine insoluble (GI) fraction, 100 μl of the guanidine stock was combined with the original tissue pellet. Both GS and GI fractions were subsequently incubated at room temperature for 1 h and were mixed every 15 min. All samples were stored at −80 °C prior to analysis. Quantification of Aβ40 and Aβ42 in the GS, GI, and plasma fractions was determined using an ELISA for human Aβ40 and Aβ42 (Invitrogen, USA) and expressed as ng of Aβ per μg total protein. The left hemisphere was used to examine the levels of soluble LRP1 in the brain. Here, the hemisphere was homogenized using a Dounce homogenizer in ice cold in HBSS using the same procedure as the in vivo LRP1 shedding studies above. LRP1 levels in the soluble brain fraction and plasma were assessed using a mouse ELISA for LRP1 (Cedar Lane Labs, USA). Soluble brain LRP1 was normalized to the total protein content in the brain homogenate as determined by the BCA protein assay (Thermo Scientific, USA). Soluble brain LRP1 levels were expressed as ng of LRP1 per mg protein. Plasma LRP1 levels were expressed as ng/ml. In addition, sAPPα levels were examined in the brain homogenate of these same animals using an ELISA for sAPPα (IBL international, USA). The sAPPα levels were normalized to the total protein content in the brain homogenate as determined by the BCA protein assay and expressed as ng of sAPPα per mg protein.

### Statistical analysis

Statistical analyses were performed using a one-way ANOVA followed by Tukey’s post hoc analysis (GraphPad Prism 5, GraphPad Software Inc., USA). Moreover, where only 2 groups were compared, unpaired t tests were used to evaluate statistical significance.

## Results

### LRP1 shedding and Aβ BBB transit in vitro

**Fig. 1:**
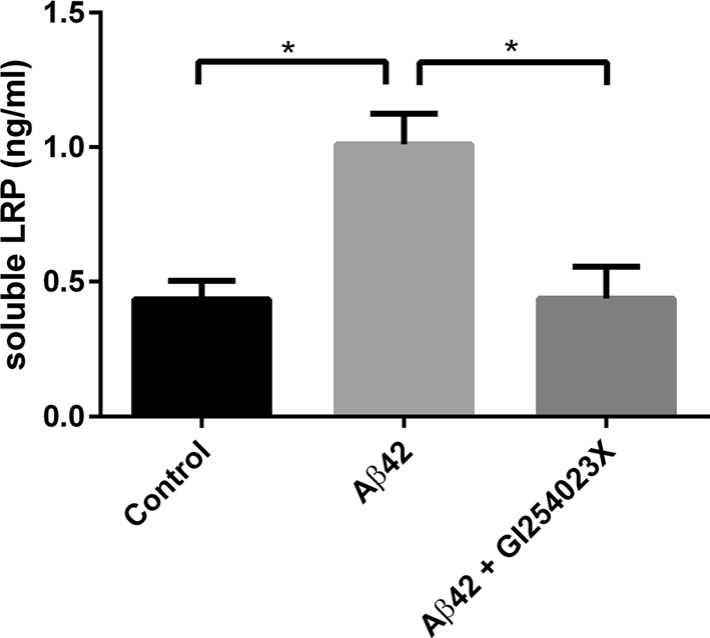
LRP1shedding in human brain endothelial cells (HBMEC) following ADAM10 inhibition. HBMEC monolayers were exposed to human Aβ42 (2 μM) alone, Aβ42 (2 μM) and the ADAM10 inhibitor GI254023X (1 μM) for 48 h at 37 °C. LRP shedding was assessed by examining LRP content in the extracellular media using an LRP1 ELISA. Values represent mean ± SEM (n = 3) and are expressed as ng of extracellular LRP1 per ml of media. *p < 0.05 as determined by one-way ANOVA followed by Tukey’s post hoc analysis

**Fig. 2:**
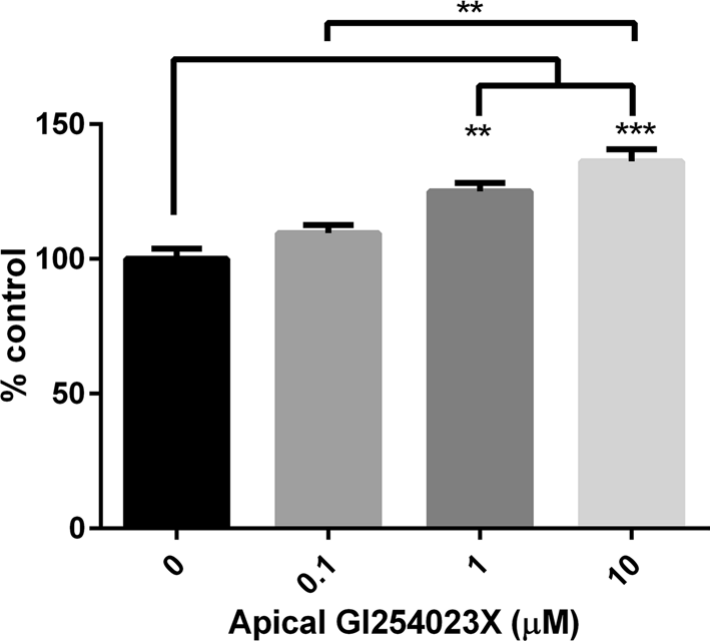
Fluorescin-Aβ42 transit across an in vitro BBB model following ADAM10 inhibition. Fluorescin-Aβ42 (2 μM) was exposed to the basolateral (“brain”) side of the in vitro BBB model, while various concentrations of the ADAM10 inhibitor GI254023X (0, 0.1, 1, and 10 μM) were exposed to the apical (“blood”) compartment. Following incubation at 37 °C, samples were collected from the basolateral compartment at 60 min to assess the basolateral-to-apical transcytosis of fluorescin-Aβ42 across the BBB model. Values represent mean ± SEM (n = 3) and are expressed as the percentage change from control conditions. *p < 0.05; **p < 0.01; ***p < 0.001 as determined by one-way ANOVA followed by Tukey’s post hoc analysis

Exposure of HBMECs to Aβ42 induced a significant increase in the extracellular shedding of LRP1 compared to control conditions (twofold) (Fig. 1). Treatment with the ADAM10 inhibitor GI254023X completely abrogated the effect of Aβ42 on shedding as extracellular LRP1 levels in the presence of GI254023X were the same as that observed under control conditions. The transcytosis of fluorescein-Aβ across an in vitro BBB model was increased following GI254023X treatment in a dose dependent manner (Fig. 2). Significant increases were observed at concentrations higher than 1 μM GI254023X showing effects of 1.25-fold or greater. Of note, no significant difference was observed between control conditions and any of the GI254023X concentrations on the BBB permeability of the paracellular marker Lucifer yellow-dextran, indicating the treatment conditions did not impact BBB integrity (data not shown).

### Brain LRP1 shedding and Aβ BBB clearance in vivo

**Fig. 3:**
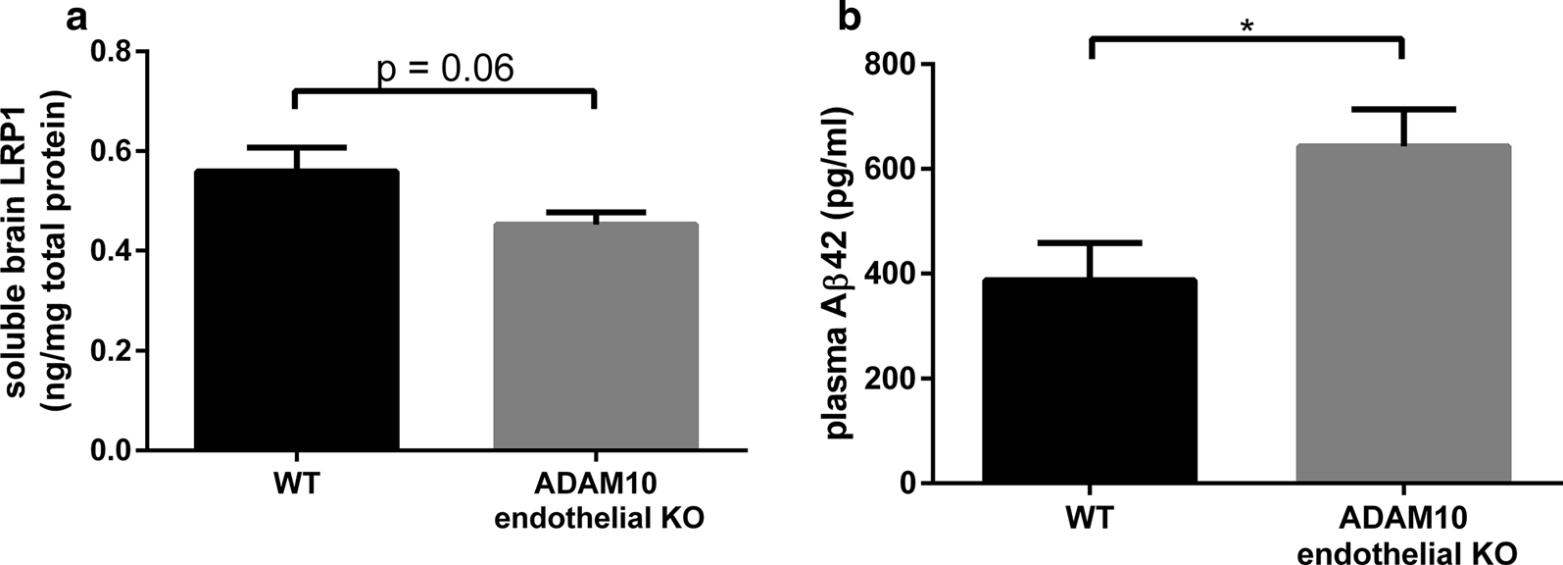
LRP1 shedding in the brain and Aβ clearance across the BBB. Human Aβ42 (1 mM) was intracranially injected into ADAM10 endothelial KO and wild-type mice. Ten minutes after the injection, the brain and plasma were collected. The soluble fraction of the brain was probed for **a** LRP1 using an ELISA, while the plasma was probed for **b** Aβ42 using an ELISA. Values represent mean ± SEM (n = 8) and are expressed as amount of LRP1 per mg total protein or amount of Aβ42 per ml. Statistical analyses were performed using an unpaired t test

LRP1 levels in the soluble brain fraction of ADAM10 endothelial KO mice were lower than that observed in wild-type animals (Fig. 3a), although this effect did not achieve statistical significance. However, Aβ clearance across the BBB was significantly greater in ADAM10 endothelial KO mice compared to wild-type animals resulting in an increase of approximately 1.75-fold (Fig. 3b).

### ADAM10 inhibition in PSAPP mice

**Fig. 4:**
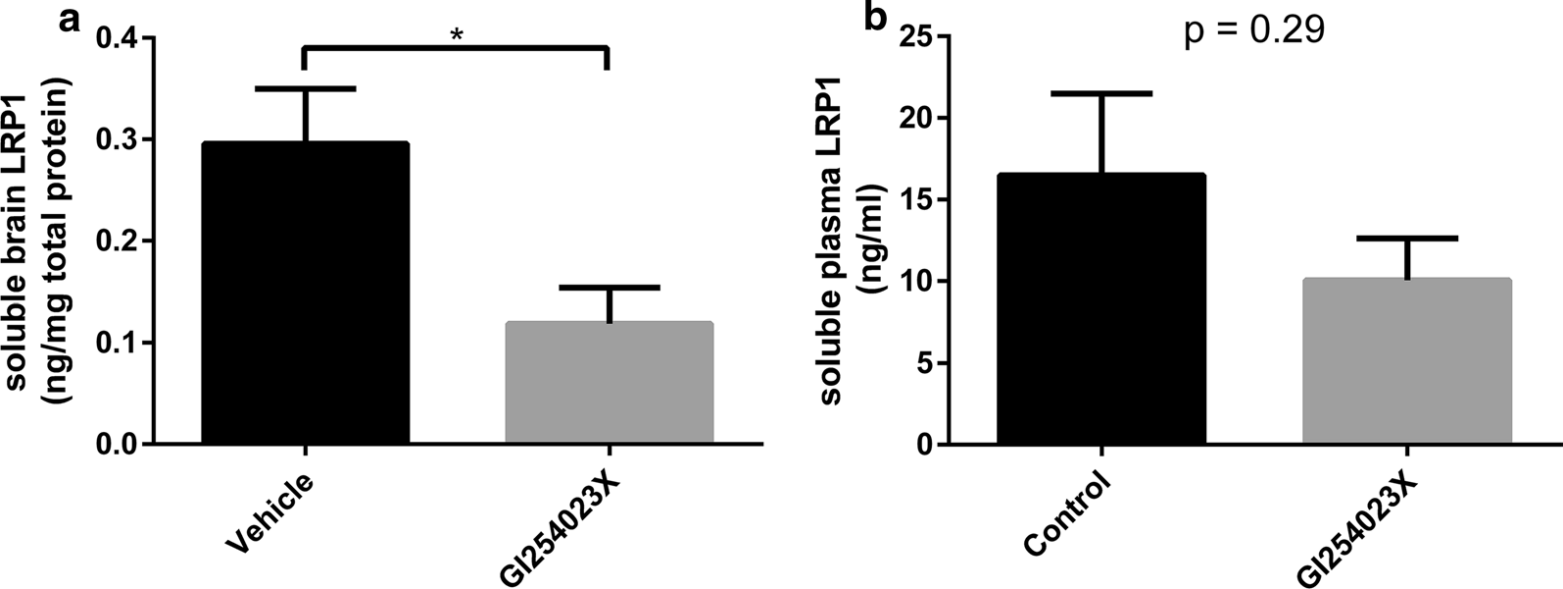
LRP1 shedding brain and plasma following ADAM10 modulation in a mouse model of AD. The ADAM10 inhibitor GI254023X (200 mg/kg) or vehicle (DMSO) was administered via intraperitoneal injection once per day for five consecutive days to PSAPP mice (35 weeks of age). One hour after the final injection, the brains and plasma was collected and the soluble brain fraction **a** and the plasma **b** was probed for LRP using an ELISA. Values represent mean ± SEM (n = 8–9 [soluble brain LRP1] or 5 [soluble plasma LRP1]) and are expressed as the amount of LRP per mg total protein or per ml. *p < 0.05 as determined by an unpaired t test

**Fig. 5:**
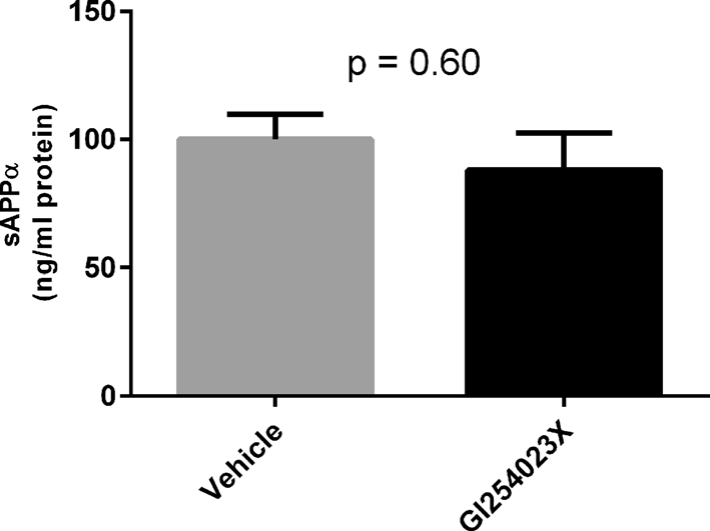
Levels of sAPPα in the brain following ADAM10 modulation in a mouse model of AD. PSAPP mice (35 weeks of age) were treated in an acute 5-day treatment paradigm with ADAM10 inhibitor GI254023X (200 mg/kg) or vehicle (DMSO) which were administered via intraperitoneal injection. One hour after the final treatment, the brains were collected and sAPPα in the whole brain homogenate was probed using an sAPPα ELISA. Values represent mean ± SEM (n = 8–9) and are expressed as the amount of sAPPα per mg total protein. Statistical significance determined by an unpaired t test

**Fig. 6:**
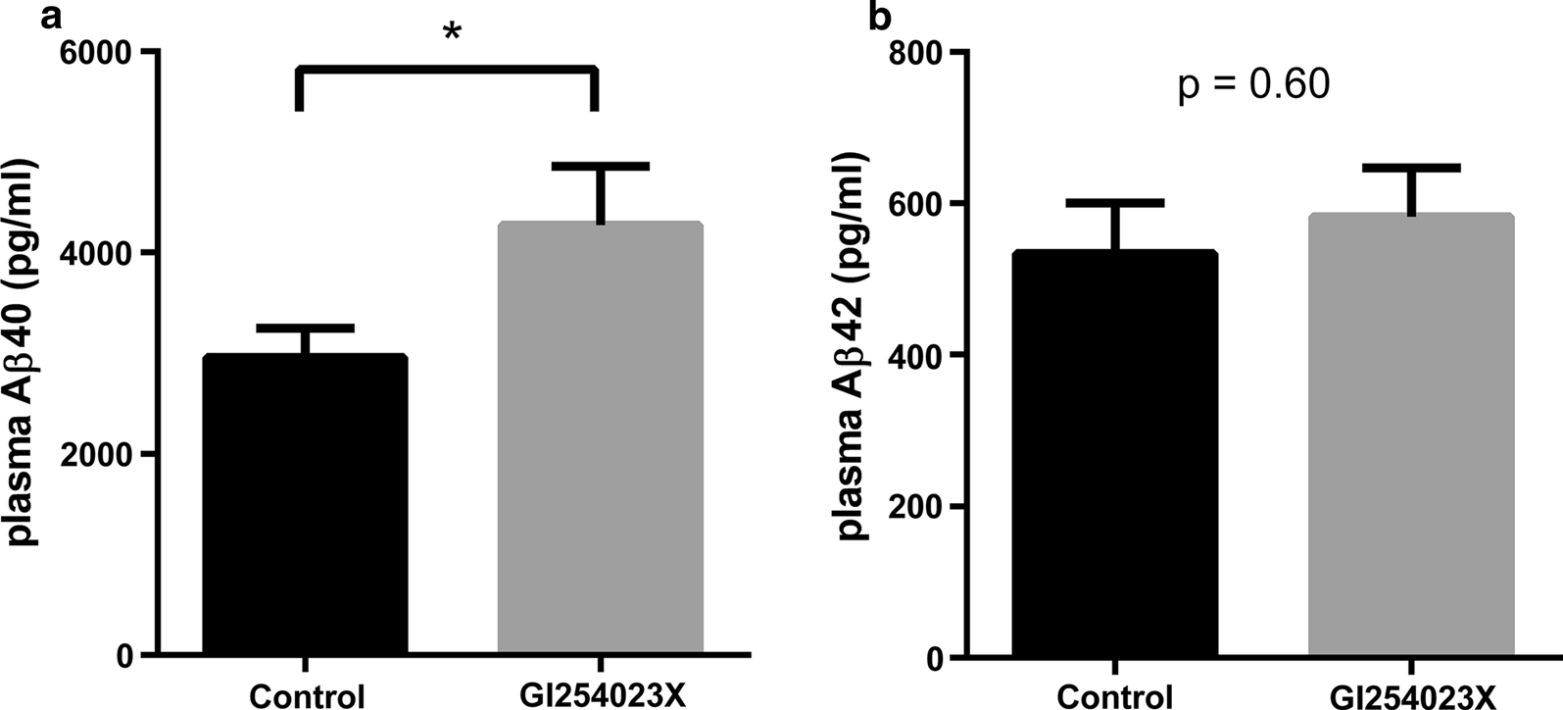
Aβ plasma levels following ADAM10 modulation in a mouse model of AD. The ADAM10 inhibitor GI254023X (200 mg/kg) or vehicle (DMSO) was administered via intraperitoneal injection once per day for five consecutive days to PSAPP mice (35 weeks of age). One hour after the final injection, the plasma was collected and probed for **a** Aβ40 and **b** Aβ42 by ELISA. Values represent mean ± SEM (n = 8–9) and are expressed as the amount of Aβ40 or Aβ42 per ml of plasma. Statistical analyses were performed using an unpaired t test

**Fig. 7:**
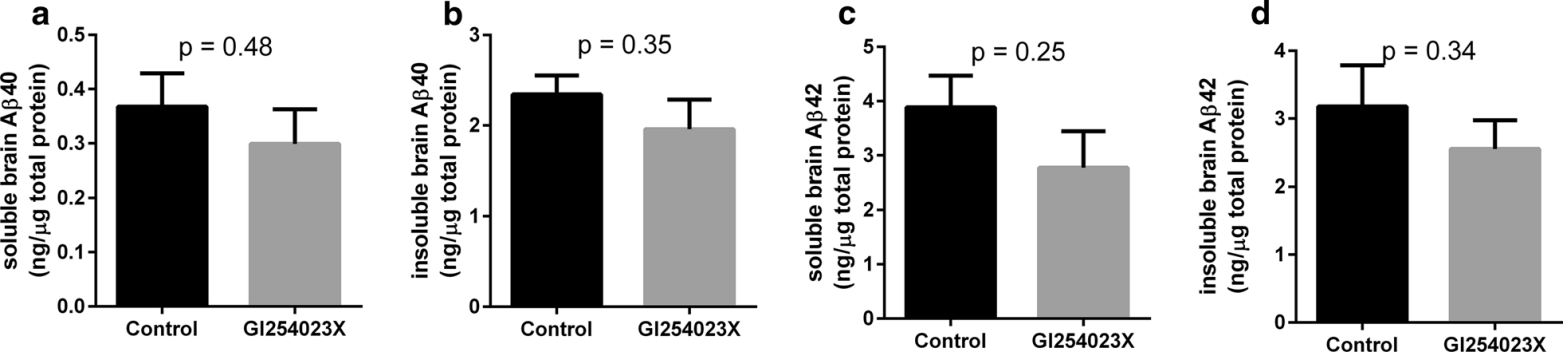
Brain Aβ levels following ADAM10 modulation in a mouse model of AD. Following a 5 day treatment of PSAPP mice (35 weeks of age) with the ADAM10 inhibitor GI254023X (200 mg/kg) or vehicle (DMSO), levels of soluble and insoluble Aβ40 and Aβ42 were measured in the brain using an Aβ40 or Aβ42 ELISA (**a**–**d**). Values represent mean ± SEM (n = 8–9) and are expressed as the amount of Aβ40 or Aβ42 per μg of total protein. Statistical analyses were performed using an unpaired t test

To evaluate the impact of ADAM10 inhibition on Aβ tissue levels and LRP1 shedding in the brain in an AD animal model, PSAPP mice were treated with the ADAM10-selective inhibitor GI254023X. The level of soluble LRP1 in the brain was significantly lower in the GI254023X-treated mice compared to the vehicle control animals (a reduction of 60 %) (Fig. 4a). In the periphery, LRP1 levels in the plasma were not significantly different when comparing treated and control animals (Fig. 4b). To assess the effect of ADAM10 inhibition on the α-secretase cleavage of APP, the levels of sAPPα in the brain were examined following GI254023X treatment. No significant difference in the level of sAPPα in the brain was detected between GI254023X-treated mice and the control group (Fig. 5). To investigate the impact of ADAM10 inhibition on the levels of Aβ40 and Aβ42 in the plasma and whole brain homogenate of PSAPP mice. GI254023X administration significantly increased the levels of Aβ40 in the plasma (1.45-fold) compared to vehicle-treated mice, while the effect of GI254023X on Aβ42 plasma levels was not significant (Fig. 6a, b). In addition, treatment with the ADAM10 inhibitor reduced both soluble and insoluble Aβ40 (Fig. 7a, b) (1.15- and 1.20-fold respectively) and Aβ42 (Fig. 7c, d) (1.25 and 1.20-fold respectively) levels in the brain compared to vehicle-treated animals, although these values did not reach statistical significance. It should also be noted that this GI254023X treatment regimen was well tolerated as the treated animals did not display any overt changes in appearance, behavior, or weight loss.

## Discussion

Dysfunction within the cerebrovascular system is now recognized as a major contributory factor in the development of AD [28]. It has been suggested that the excessive accumulation of Aβ in the AD brain may be due to aberrant Aβ elimination [29, 30] as several studies have identified reduced brain Aβ clearance in AD patients and AD animal models [5, 7]. More specifically, an important component in the removal of Aβ from the brain involves cerebrovascular transport across the BBB. Prior studies have shown that reduced levels of the receptors that transport Aβ in brain endothelia, such as LRP1, results in decreased Aβ clearance across the BBB, elevated Aβ burden in the brain, and aggravated memory and learning deficits [11, 29]. In normal aging and AD, LRP1 expression is diminished in the brain vasculature [10, 31–33] and correlates with Aβ accumulation in the brain and cerebrovasculature [13, 32]. In addition, the expression of LRP1 can be affected by a variety of upstream events such as the promoter methylation status, which controls the generation of LRP1 mRNA [34]. Previously it has been shown that promoting LRP1 expression enhances the cellular uptake of Aβ [35] and facilitates Aβ clearance from the brain extracellular space [36]. Our previous work shows LRP1 is susceptible to ectodomain shedding, a process that inactivates the transport capabilities of the receptor, which results in reduced Aβ clearance across the BBB [17]. The current studies evaluated a treatment strategy targeting LRP1 shedding in the brain as a novel means to promote Aβ BBB clearance and attenuate Aβ accumulation in the AD brain.

Previous investigations have implicated the α-secretase enzyme, ADAM10, in the ectodomain shedding of LRP1 [18, 37]. In the present studies, we found that inhibition of ADAM10 effectively reduced LRP1 shedding and increased Aβ transit across an in vitro model of the BBB. This was supported by our in vivo studies in which ADAM10 endothelial KO mice displayed less LRP1 shedding in the brain in conjunction with increased Aβ clearance across the BBB compared to wild-type animals. However, the reduced shedding of brain LRP1 in the ADAM10 endothelial KO group did not reach statistical significance when compared to wild-type mice. This may not be all that unexpected as there are number of cell types in the brain that express LRP1 in addition to brain endothelia. As our analytical approach assessed LRP1 levels in the entire soluble fraction of the brain, endothelial-specific changes in LRP1 shedding may be difficult to capture with so many other cells types contributing to the soluble pool of LRP in the brain. Nevertheless, our findings in vitro and in vivo demonstrate that modulation of the ADAM10 enzyme minimizes LRP1 shedding and facilitates Aβ clearance across the BBB.

As we identified a role for ADAM10 in mediating Aβ clearance across the BBB, we next examined the impact of ADAM10 inhibition on Aβ tissue levels in an animal model of AD. Using an acute 5-day treatment paradigm with the ADAM10-selective inhibitor GI254023X in PSAPP mice, we observed a substantial decrease in LRP1 shedding in the brain compared to vehicle-treated animals. Moreover, as previous studies have reported that soluble LRP1 levels in the plasma can facilitate Aβ removal from the brain by acting as a peripheral sink [12], we investigated LRP1 levels in the plasma following GI254023X administration. Soluble LRP1 levels in the plasma were found not to be significantly different between treated and control animals. However, we did find that The decrease in soluble LRP1 in the brain following treatment with GI254023X coincided with a significant increase in plasma Aβ40 levels. As such, we propose that inhibition of ADAM10 facilitates Aβ40 transit from the brain to the periphery (via reduced LRP shedding at the BBB), resulting in the elevated Aβ40 plasma levels we observed. However, no significant changes in plasma Aβ42 levels were detected following GI254023X treatment. A potential explanation for the disparity we observed in the BBB clearance between Aβ40 and Aβ42 may be due to differences in the transport rates for these species. A recent report indicated LRP1 preferentially clears Aβ40 over Aβ42 [11] and prior studies have shown that the rate of Aβ40 transport across the BBB is more than twice that observed for Aβ42 [8, 38]. Nevertheless, the increased levels of Aβ40 in the plasma following ADAM10 inhibition suggest this treatment strategy may be used to facilitate the transit of Aβ from the brain to the periphery.

To examine the impact of ADAM10 inhibition on Aβ levels in the brain, we examined both soluble and insoluble Aβ following GI254023X treatment in PSAPP animals. While we observed reductions in both soluble and insoluble Aβ40 and Aβ42 in the brain following GI254023X treatment, none of these effects were statistically significant. It is unclear why this treatment paradigm did not modulate Aβ levels in the brain more effectively. Both our prior work [17] and our current findings demonstrate a strong relationship between brain LRP1 shedding and Aβ clearance across the BBB. GI254023X treatment in the PSAPP mice reduced LRP1 shedding in the brain by 60 %, however, this effect did not translate to significant changes in Aβ levels in the brain. It may be that such an acute treatment paradigm (5 days) is not sufficient to demonstrably lower Aβ levels in the brain, and that a more chronic treatment paradigm is necessary. Another possible explanation for our observations is that LRP1 expression is known to be lower in AD patients [13, 39] and AD animals, including the PSAPP mice used in the current studies [40]. Exposure of brain vascular cells to high levels of Aβ for a prolonged period has previously been shown to reduce the expression of LRP1 [39]. In our study, the PSAPP mice were tested at an age when extensive Aβ pathology is present [22, 41]. As such, the total LRP1 population may be depleted to such an extent that any improvements in BBB LRP1 shedding to promote Aβ elimination would still prove insufficient. Therefore, this therapeutic approach may be more impactful if used earlier in the disease process when a greater density of LRP1 receptors is available for therapeutic targeting. Further evaluation of this treatment protocol and the feasibility of targeting LRP1 sheddase enzymes in AD are certainly warranted.

A primary concern in targeting an enzyme like ADAM10, especially in AD, is the potential impact on other substrates that are metabolized by ADAM10. ADAM10 is one of the α-secretases which processes APP through the non-amyloidogenic pathway, resulting in the formation of sAPPα while at the same time avoiding the production of Aβ peptides. As such, inhibition of this pathway could facilitate Aβ synthesis and potentially exacerbate Aβ burden in the AD brain. Our data suggests this is not the case, as Aβ levels in the brain did not increase upon ADAM10 inhibition, but in fact decreased. To ascertain whether GI254023X treatment influenced the α-secretase pathway specifically, we measured sAPPα levels in the brain and found no difference between GI254023X-treated animals and the vehicle control group. These data indicate ADAM10 can be modulated to reduce LRP1 shedding in the brain without affecting the α-secretase cleavage of APP. One explanation for this may be the presence of other α-secretase enzymes, which process APP when ADAM10 is diminished or absent. To this point, it was previously found that sAPPα formation was preserved in fibroblast cells derived from ADAM10-deficient animals [42]. It has also been reported that other members of the α-secretase family such as ADAM9 and ADAM17 are able to compensate for reductions in ADAM10 activity [42, 43]. Alternatively, other studies have reported a significant change in sAPPα production or increase in Aβ when ADAM10 was absent or diminished, suggesting a lack of compensation by other α-secretases [19, 44–46]. Nevertheless, our studies demonstrate that targeting the ADAM10 enzyme can effectively reduce LRP1 shedding in the cerebrovasculature without impacting APP proteolysis.

## Conclusions

Previously, our work demonstrated a strong inverse correlation between LRP1 shedding in the brain and Aβ clearance across the BBB. In the present studies, we show that modulation of the ADAM10 enzyme can effectively reduce LRP1 shedding and promote Aβ transport out of the brain. In an AD mouse model, acute treatment with an ADAM10 inhibitor substantially lowered LRP1 brain shedding and increased the appearance of Aβ40 in the plasma, indicating enhanced Aβ transit from the brain to the periphery. Alternatively, while ADAM10 inhibition decreased both Aβ40 and Aβ42 brain levels in the AD mice, these values were not statistically significant, indicating a more chronic treatment paradigm may be necessary to observe demonstrable changes in Aβ brain burden. Nevertheless, while further interrogation of this therapeutic approach is necessary, our findings indicate LRP1 sheddases can be targeted to facilitate Aβ elimination from the brain, providing a novel therapeutic strategy to mitigate Aβ accumulation in the AD brain.

## Abbreviations

ADAM10: a desintegrin and metalloproteinase domain containing protein 10
AD: Alzheimer’s disease
APP: amyloid precursor protein
Aβ: beta-amyloid
BBB: blood–brain barrier
CAA: cerebral amyloid angiopathy
LRP1: low density lipoprotein receptor-related protein 1
